# Atypical Presentation of Small Bowel Neuroendocrine Carcinoma Leading to Acute Obstruction

**DOI:** 10.7759/cureus.84661

**Published:** 2025-05-23

**Authors:** Aravind Kumar, V Ramlakshmi, Samir Ahmad, Alexander Mecheri Antony

**Affiliations:** 1 General Surgery, Sree Balaji Medical College and Hospital, Chennai, IND

**Keywords:** acute intestinal obstruction, carcinoid syndrome, gasterointesinal tract, neuroendocrine carcinoma(nec), neuro endocrine tumor

## Abstract

Rare neoplasms called neuroendocrine tumors (NETs) develop from intestinal enterochromaffin cells. They commonly produce symptoms through the secretion of serotonin along with other vasoactive peptides, resulting in carcinoid syndrome, characterized by cutaneous flushing and diarrhea. NETs are a very aggressive type of cancer, for which prognostic factors are lacking. They are also rarely found in males and young adults. Carcinoid tumors make up an atypical and intricate disease spectrum with various clinical features. The combination of etoposide and carboplatin can enhance overall survival in complex WHO stage 3 neuroendocrine carcinoma with regional lymph node involvement and a generally poor prognosis. However, in the absence of distant metastasis and with a relatively fair performance index, this treatment may be more effective. In this instance, we report a neuroendocrine tumor case that presented unusually as an acute intestinal obstruction. The patient had resection and anastomosis of the small bowel of the affected region along with the surrounding mesentery.

## Introduction

With an estimated yearly incidence of 1-2 per 100,000, neuroendocrine tumors (NETs) are rare and often slow-growing neoplasms. These tumors can be seen in a variety of primary tumor locations because they originate from the endocrine cells of the body's diffuse endocrine system (DES). The WHO introduced a new classification system in the year 2000 that included the terms (neuro-)endocrine tumor or cancer [1]. Enterochromaffin cells in the intestine are the source of NETs. These cells produce various hormones and neurotransmitters and are a component of the diffuse neuroendocrine system [2]. NETs are unique in their ability to secrete biologically active substances, including serotonin and other vasoactive peptides. They produce neuroamines and peptides that cause certain clinical disorders, like carcinoid syndrome [3]. It is commonly responsible for the symptoms of carcinoid syndrome, which include cutaneous flushing, diarrhea, bronchospasm, and, in rare cases, carcinoid heart disease [4]. Carcinoid syndrome is more commonly seen in metastatic disease, particularly when the tumor has spread to the liver. Associated noncarcinoid tumors were common alongside small intestine (29.0%), colonic (20.0%), gastric (20.5%), and appendiceal (18.2%) carcinoids. Cecal (81.5-83.2%) and pancreatic (71.9-81.3%) carcinoids had the largest percentages of nonlocalized lesions, while the rectal (81.7%), gastric (67.5%), and bronchopulmonary (65.4%) carcinoids all had the highest percentages of localized disease [5].

## Case presentation

**Figure 1:**
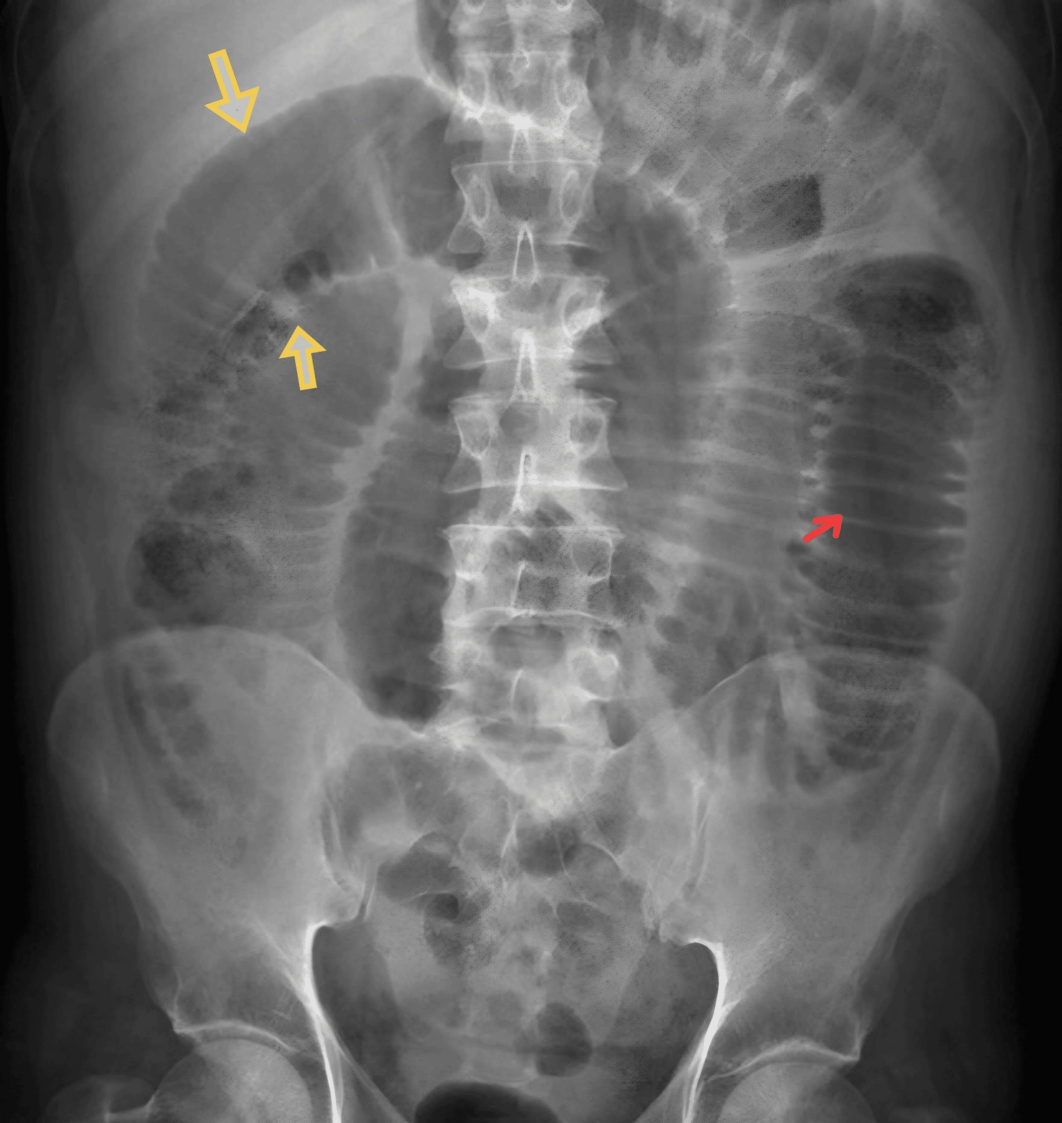
X-ray erect abdomen showing multiple dilated bowel loops suggestive of small bowel obstruction In small bowel obstruction, the centrally positioned gas-filled and distended bowel loops (marked by yellow arrows) are noted. The white lines traversing the full width of the bowel are valvulae conniventes (marked by red arrows), which are characteristic of the small bowel.

**Figure 2:**
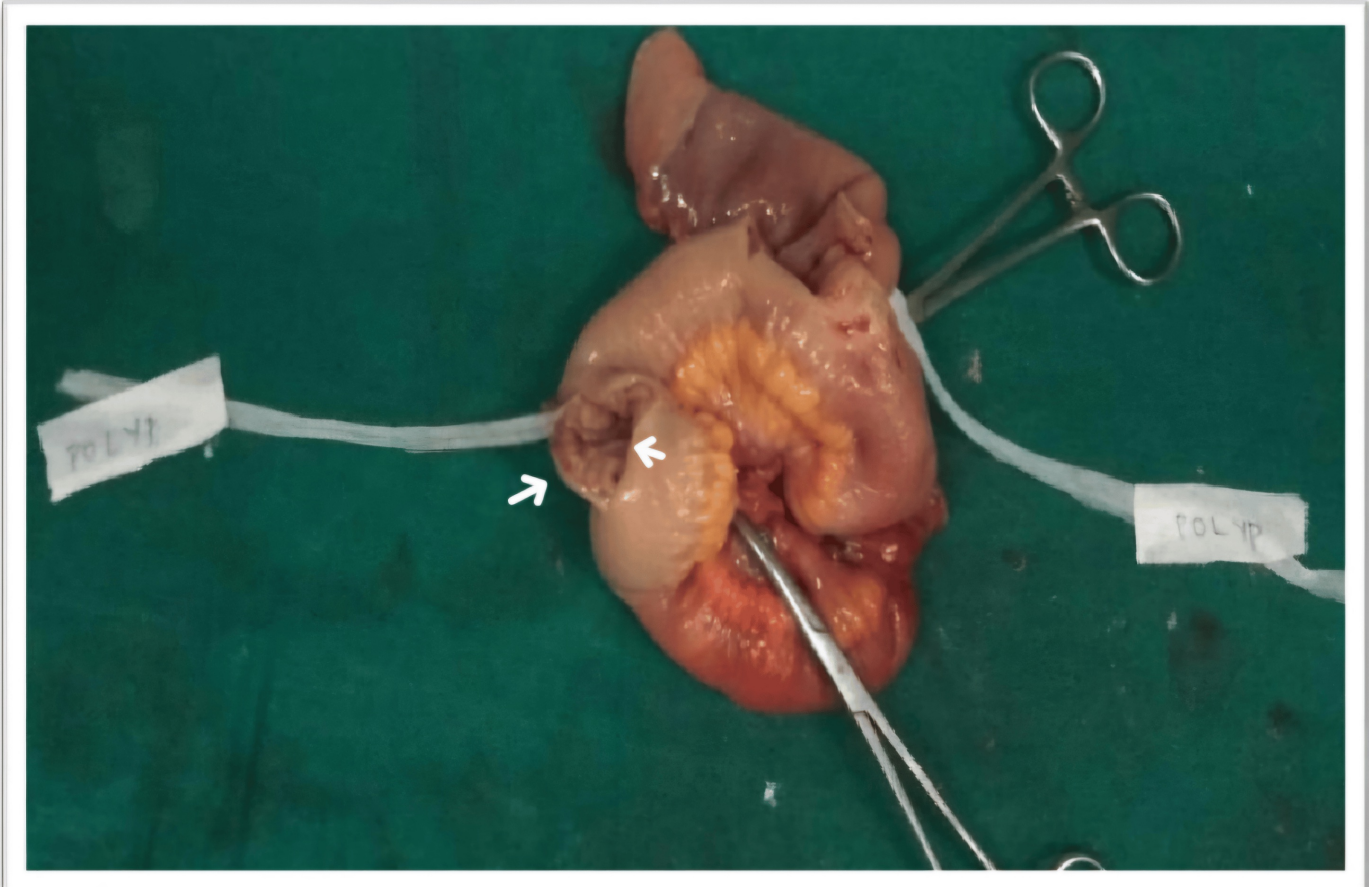
Resected segment of congested and edematous small bowel showing two polyps (noted with arrows) The polyps, each measuring approximately 2 × 2 × 1 cm, were located about 20 cm proximal to the ileocecal junction (ICJ). The surrounding bowel loops were congested and edematous, with cicatrized mesentery and a stricture in the ileum at the same level.

**Figure 3:**
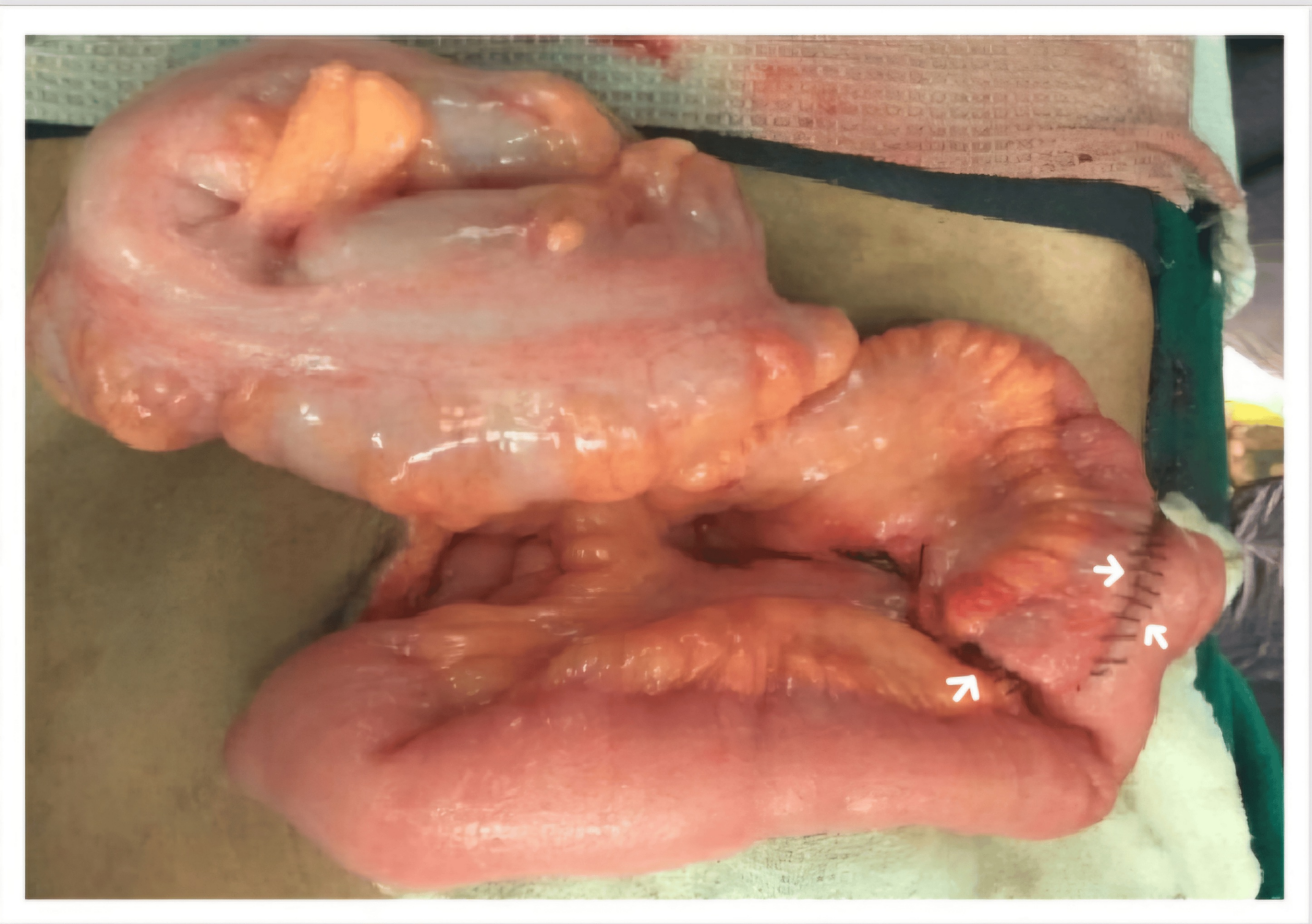
Resection and anastomosis of the small bowel of the affected region of the bowel along with the surrounding mesentry The affected segment of the bowel was resected along with the mesentery, and end-to-end anastomosis was performed.

**Figure 4:**
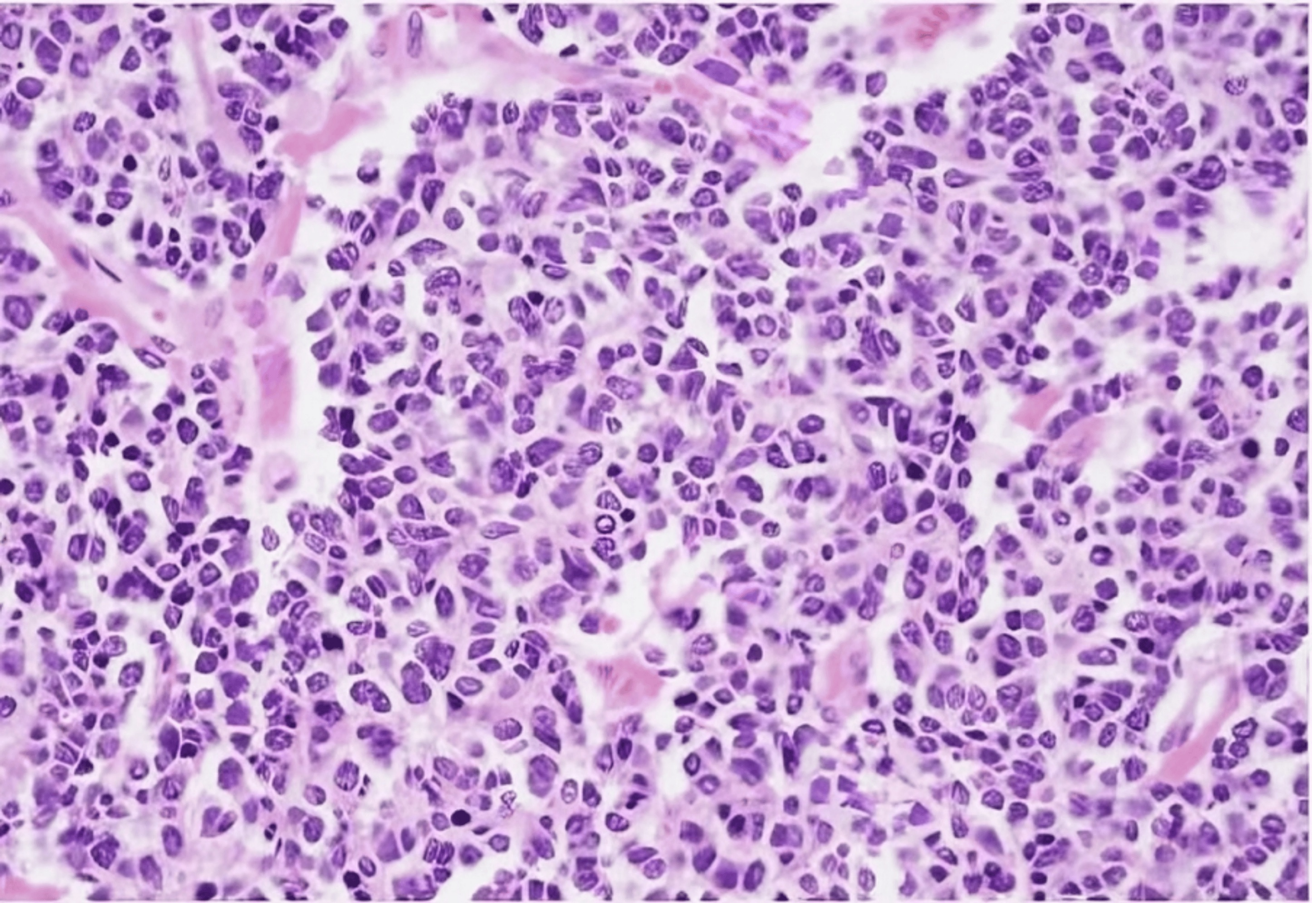
Neuroendocrine tumor histopathology showing monotonous cells with round to oval nuclei and finely speckled “salt and pepper” chromatin No mitotic activity is observed in this case.

We report a unique case of a 52-year-old male who presented with abdominal pain and frequent episodes of vomiting for two days. An examination of the abdomen showed localized guarding over the lower abdomen and discomfort in the hypogastrium, suprapubic area, right iliac fossa, and left iliac fossa. Multiple dilated bowel loops were shown in the X-ray of the abdomen (Figure 1); small bowel obstruction with intussusception was indicated by the ultrasound scan. We planned an emergency laparotomy for the patient. The intraoperative findings revealed two polyps measuring approximately 2 × 2 × 1 cm, located approximately 20 cm proximal to the ileocecal junction (ICJ) (Figure 2). The loops of the intestine were swollen and had poor blood flow, with scarred tissue in the mesentery and a narrowing in the ileum 20 cm before the ileocecal junction. The affected segment of the bowel was resected with mesentery, and end-to-end anastomosis (Figure 3) was performed. The histopathological examination (HPE) (Figure 4) confirmed the presence of an NET in the small bowel, classified as T2N1M0, with a Ki-67 index of 70%, and the edges of the removed tissue were clear of cancer (R0). Postoperatively, the patient was managed with appropriate intravenous antibiotics and other supportive medications and was mobilized on Day 1. He was started on incentive spirometry and chest physiotherapy. Ryle's tube and Foley's catheter were removed on Day 2, and he was started on sips followed by a liquid diet on Day 3, which was well tolerated. He was started on a semisolid diet on Day 5, which was also well tolerated, and the patient was discharged on Day 7. The patient remained in regular follow-up and was subsequently started on adjuvant chemotherapy with cisplatin and etoposide.

## Discussion

NETs are predominantly located in the bronchi, the lungs, as well as the gastrointestinal (GI) tract. In the GI tract, they involve the appendix, the ileum, the colon, the stomach, and the rectum. NETs are a type of tumor that can look and behave differently under a microscope and in chemical tests, and they make substances like serotonin and vasoactive peptides, which can lead to carcinoid syndrome [6]. Diarrhea, skin flushing, and the development of carcinoid heart disease, which progresses from narrowing of the pulmonary valve to leaking of the tricuspid valve to narrowing of the tricuspid valve, are key signs of carcinoid syndrome [7]. The lungs, bronchi, as well as the gastrointestinal tract, are the most prevalent locations for neuroendocrine tumors, which have a variety of histological and biochemical profiles [8]. Diagnosis involves the use of biomarkers and advanced imaging techniques. Management typically includes surgical intervention, chemotherapy, and targeted therapies [9]. The results of previous research are supported by the Ki-67 index, which is a strong and independent predictor. The primary technique for verifying the neuroendocrine features of the tumor is immunohistochemistry for vesicular marker proteins [10].

More broadly, a high lactate dehydrogenase (LDH) level is recognized as a poor prognostic indicator in various cancers, including lung and breast cancer. Additionally, two studies found elevated LDH serum levels in neuroendocrine carcinoma (NEC): one in the lungs and the other in colorectal cancer [11]. An elevated LDH level can be attributed to tumor cells' dependence on enhanced glycolysis, leading to higher lactate production rather than aerobic respiration in the mitochondria, even when oxygen is plentiful. This phenomenon is referred to as the Warburg effect. The proposed treatment strategy of surgical resection and chemotherapy can be considered evidence-based for the treatment of advanced or atypically presenting NETs. We practice this by combining palliative chemotherapy with surgery to debulk the tumor mass. These effective treatments include the radiolabeled somatostatin analogue lutetium-177 (177Lu)-dotatate, the mechanistic target of rapamycin inhibitor everolimus, the multitargeted receptor tyrosine kinase inhibitor sunitinib, the vascular endothelial growth factor antibody bevacizumab, and new combinations of previously approved treatments. NETs are heterogeneous neoplasms with a broad spectrum of clinical manifestations. Biomarkers include urine 5-hydroxyindoleacetic acid (5-HIAA), synaptophysin on biopsy, and serum chromogranin A. The imaging tests needed are a computed tomography (CT) scan, somatostatin-receptor scintigraphy (Octreoscan™), endoscopic ultrasonography (mainly for duodenal and pancreatic NET), magnetic resonance imaging (MRI), or DOT A-TOC FDG/PET.

Principles of management 

Segmental resection is the method used in treating small bowel tumors that are less than 1 cm. Tumors measuring one centimeter or more require the removal of the surrounding mesentery as well. Irrespective of tumor size, a right hemicolectomy is the recommended operation for the terminal ileum. For rectal lesions, endoscopic resection is used for lesions less than 1 cm. Tumors measuring one centimeter or larger receive treatment through anterior resection.

The chemotherapy regimen combines 5-fluorouracil (5-FU) with streptozocin and cisplatin, while pasireotide, a somatostatin analogue, and peptide receptor radionuclide therapy (PRRT) employ somatostatin analogues conjugated with radioactive isotopes. Targeted therapies include telotristat etiprate (tryptophan hydroxylase inhibitor), bevacizumab (anti-VEGF), sunitinib (tyrosine kinase inhibitor), and everolimus (motor inhibitor).

## Conclusions

Since NETs can present in various forms and may have hormonal activity, diagnosis and management can be challenging. This case highlights the significance of taking NETs into account while making a differential diagnosis in a case of acute intestinal obstruction. It also underscores the importance of complete surgical and medical therapy to achieve the best outcomes. Such an approach, combining chemotherapy with surgical resection of the tumor, is in concordance with current management guidelines for advanced or atypical neuroendocrine tumors. NETs present in various ways. In this regard, it is imperative to carry out investigations and evaluate the stage of the disease. Therefore, it is essential to emphasize the value of an integrated approach to the therapy of patients with this condition and to include NET in the list of diseases that should be considered during the differential diagnosis of acute intestinal obstruction.
